# Betulinic Acid and Betulin Suppress Melanoma Growth by Modulating Apoptosis and Autophagy via PI3K/AKT/mTOR and MAPK Pathways

**DOI:** 10.3390/ijms27020576

**Published:** 2026-01-06

**Authors:** Yingying Zhang, Meng Yuan, Quan Xu, Jun Lin, Pei Lin

**Affiliations:** School of Life Sciences and Health Engineering, Jiangnan University, Wuxi 214122, China; 6233302028@stu.jiangnan.edu.cn (Y.Z.); 6233302026@stu.jiangnan.edu.cn (M.Y.); 6253302059@stu.jiangnan.edu.cn (Q.X.); junlin@jiangnan.edu.cn (J.L.)

**Keywords:** betulinic acid, betulin, melanoma, network pharmacology, autophagy, PI3K/AKT/mTOR

## Abstract

Malignant melanoma (MM) is a highly invasive and metastatic form of skin cancer. Betulinic acid (BA) and betulin (BE) possess pharmacological activities such as heat-clearing, detoxification, and anti-tumor effects, with BA showing potent selective cytotoxicity against melanoma cells. However, their underlying mechanisms in MM treatment remain unclear. Herein, this study systematically evaluated the anti-melanoma effects of BA and BE via integrated network pharmacology, in vitro and in vivo assays. Network pharmacology analysis revealed that BA and BE exerted anti-MM effects mainly by regulating apoptosis, angiogenesis and autophagy through the PI3K/AKT and MAPK signaling pathways. In vitro, both BA and BE inhibited colony formation and migration of B16-F10 cells, induced apoptosis by enhancing DNA damage and upregulating apoptotic protein expression, increased autophagic activity, and reduced ATP production and mitochondrial membrane potential (ΔΨm). These effects were closely associated with the inhibition of the PI3K/AKT/mTOR and MAPK pathways. Notably, BA showed stronger inhibitory effects than BE on the migration, invasion and tube formation of HUVECs. In vivo assays further confirmed that BA significantly suppressed melanoma growth in C57BL/6J mice by blocking the PI3K/AKT/mTOR and MAPK pathways. Collectively, BA and BE inhibit B16-F10 cell proliferation through the regulation of apoptosis and autophagy, with BA showing particularly promising potential as a candidate agent for MM therapy.

## 1. Introduction

The initiation and progression of malignant melanoma (MM) involve a complex, multi-step biological process influenced by genetic predisposition, environmental influences (primarily ultraviolet radiation), and alterations in molecular pathways. Among environmental risk factors, prolonged exposure to ultraviolet (UV) radiation is a major contributor to melanomagenesis [1]. UVA interacts with photosensitizing substances within cells to generate reactive oxygen species (ROS). These ROS damage both DNA and the proteins responsible for DNA repair, ultimately leading to the transformation of melanocytes into melanoma cells [2,3]. Additionally, genetic mutations are pivotal in the onset and advancement of melanoma. Among these, BRAF mutations, especially the V600E variant, are the most prevalent, accounting for approximately 90% of BRAF-mutant melanomas [4,5]. Constitutive activation of the mitogen-activated protein kinase (MAPK) signaling cascade, largely driven by BRAF mutations, is recognized as a key initiator of melanoma development [6]. Concurrently, aberrant activity in the phosphatidylinositol 3-kinase (PI3K)/AKT pathway, often associated with loss of the tumor suppressor phosphatase and tensin homolog (PTEN), plays a critical role in promoting tumor progression, therapeutic resistance, and maintaining the malignant phenotypes. Together, the PI3K/AKT and MAPK pathways form the core of disrupted signaling networks in melanoma and serve as major therapeutic targets [7,8].

During early tumorigenesis, autophagy inhibits oncogenesis by eliminating damaged organelles and misfolded proteins to preserve cellular homeostasis [9]. However, in advanced malignancies or under therapeutic stress, enhanced autophagy promotes survival by degrading toxic intracellular components and supplying energy and building blocks [10]. Notably, excessive autophagy or its crosstalk with apoptotic machinery, such as the Bcl-2-interacting protein1(Beclin-1)/B-cell lymphoma-2 (Bcl-2) interaction releasing proapoptotic factors, can ultimately induce cell death in melanoma cells [11,12]. Therefore, the strategic co-regulation of autophagy and apoptosis has emerged as a critical frontier in melanoma research.

Current treatment modalities for MM, including surgical excision, radiotherapy, chemotherapy, immunotherapy, and targeted hormonal therapy, often suffer from substantial side effects and therapeutic limitations [13,14]. Natural products have gained increasing attention as promising anticancer agents due to their structural diversity, broad bioactivity, and relatively low toxicity profiles [15]. Among these, terpenes and terpenoids demonstrated pharmacological properties, including anti-inflammation, antibacterial, antibacterial, antioxidative, and anticancer effects [16]. Betulinic acid (BA) and betulin (BE), two naturally occurring pentacyclic lupane-type triterpenoids abundantly found in the bark of *Betula platyphylla Suk* [17], exhibit pronounced selective cytotoxicity against tumor cells at both molecular and cellular levels [18,19]. Compelling evidence demonstrates BA’s pro-apoptotic efficacy across various cancer types [20,21,22], in vivo investigations indicate that it may act as a melanoma-specific cytotoxic agent [23]. BE has also demonstrated anticancer potential, characterized by high safety, low cost, and effective growth inhibition across multiple tumor models such as breast and gastric cancers, often through the promotion of apoptosis [24,25]. However, the mechanisms underlying the anti-melanoma effects of BA and BE remain unclear. This study employed network pharmacology analysis to analyze potential drug targets and pathways of BA and BE against melanoma, and systematically evaluated the mechanisms of through the in vitro and in vivo experiments.

## 2. Results

### 2.1. Network Pharmacological Analysis

**Figure 1:**
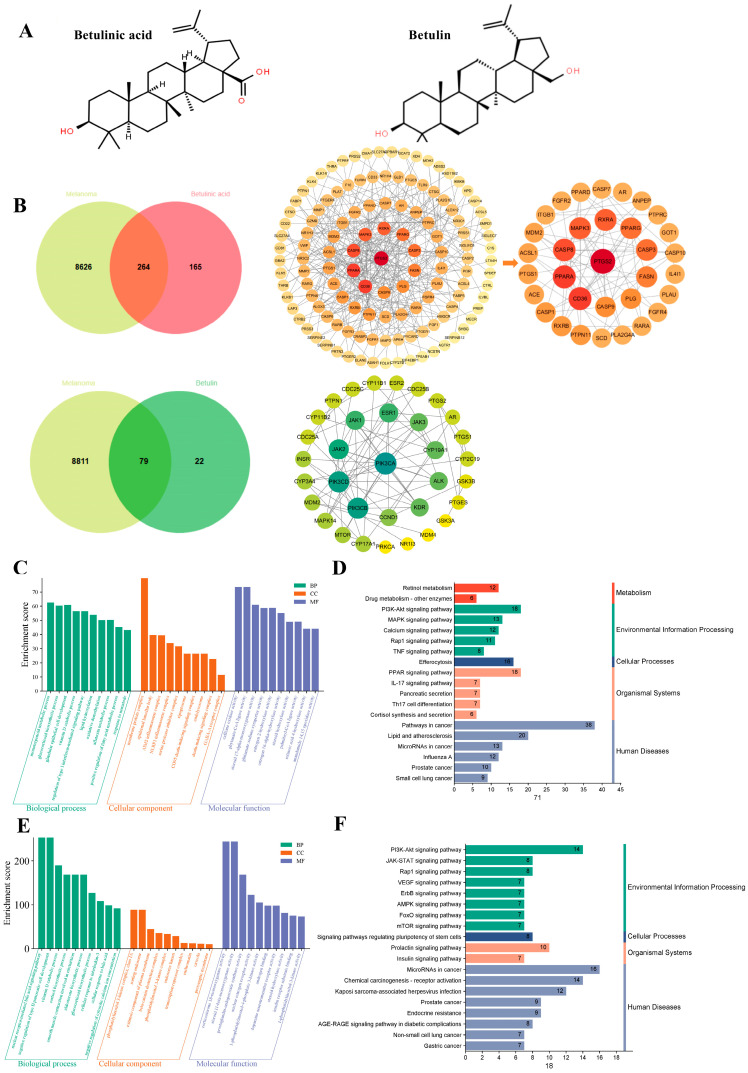
Network pharmacological analysis of BA and BE on anti-melanoma. (**A**) The chemical structure of BA and BE. (**B**) Venn diagram of BA or BE and MM associated proteins. Cluster analysis of the PPI network for cross-interacting proteins in BA, BE-MM, where deeper colors and larger node sizes indicate stronger correlations. (**C**,**D**) BA target-related GO and KEGG pathway enrichment analyses. (**E**,**F**) BE target-related GO and KEGG pathway enrichment analyses.

The overlapping targets of BA and BE with MM-associated genes were identified and visualized using a Venn diagram. A total of 264 and 79 common targets were identified for BA and BE, respectively, and subsequently analyzed via the STRING database. Visualization was performed using Cytoscape software version 3.10.2 to generate a PPI network map. After removing targets with fewer interactions, 126 and 34 potential targets were identified for BA and BE, respectively, and used for the initial network construction. The top 33 BA-associated targets, ranked by interaction degree value, were selected for final visualization (Figure 1B). These targets were primarily involved in the regulation of angiogenesis, inflammation, cell proliferation, migration, autophagy, and apoptosis, and included PPAR, MAPK3, CASP3, CASP9, PIK3C, and JAK.

To identify the biological characteristics associated with the anti-melanoma effects of BA and BE, we conducted GO and KEGG analyses on the targets within the PPI network using the DAVID database (Figure 1C–F). Among the signaling pathways enriched by the KEGG analysis, the treatment of melanoma with BA was associated with several pathways, including pathways in cancer, PI3K-AKT signaling pathway, PPAR signaling pathway, lipid and atherosclerosis, and MAPK signaling pathway. The treatment of melanoma with BE was associated with MicroRNAs in cancer, chemical carcinogenesis-receptor activation, Kaposi sarcoma-associated herpesvirus infection PI3K-AKT signaling pathway, and prolactin signaling pathway. These findings indicate that the anti-melanoma effects of BA or BE were closely related to their ability to regulate cell migration, angiogenesis, autophagy, and apoptosis.

### 2.2. The Antioxidant Capacity of BA and BE and Effects on the Proliferation of B16-F10 Cell

**Figure 2:**
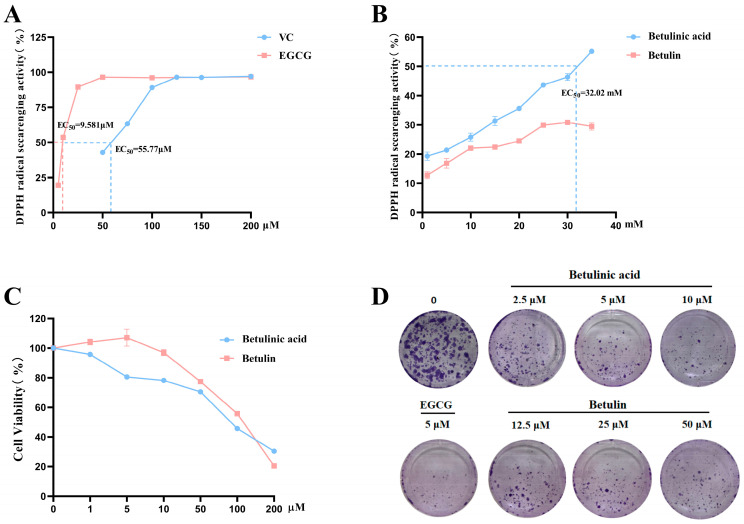
The effects of BA and BE on the proliferation of B16-F10 cells. (**A**,**B**) DPPH radicals scavenge assay of BA and BE. The dashed line in the figure indicates the median effective concentration (EC50). (**C**) Cytotoxicity of BA and BE on B16-F10 cells after 24 h treatment. (**D**) The colony formation test was measured after 24 h of BA and BE treatment (*n* = 6).

DPPH radicals scavenge was assessed to evaluate the antioxidant activities of BA and BE using vitamin C (VC) as a positive control. As shown in Figure 2A,B, both VC and EGCG exhibited free radical scavenging activities, while BA demonstrating stronger effect than BE. After treatment with BA or BE for 24 h, the viability of B16-F10 cells remained above 80% (Figure 2C). Based on these results, BA was applied at concentrations of 2.5, 5, and 10 μM, while BE was tested at 12.5, 25, and 50 μM in subsequent experiments. Colony formation assays were performed to investigate the inhibitory effects of BA and BE on B16-F10 cell proliferation. EGCG inhibited the proliferation of melanoma cells and induced apoptosis, demonstrating therapeutic potential against melanoma [26], subsequent experiments used EGCG as a positive control (Figure S1). Compared to the normal control group, both BA- and BE-treated groups formed smaller colonies in a manner dependent on the administered dose (Figure 2D).

### 2.3. BA and BE Inhibit the Migration of B16-F10 Cells

**Figure 3:**
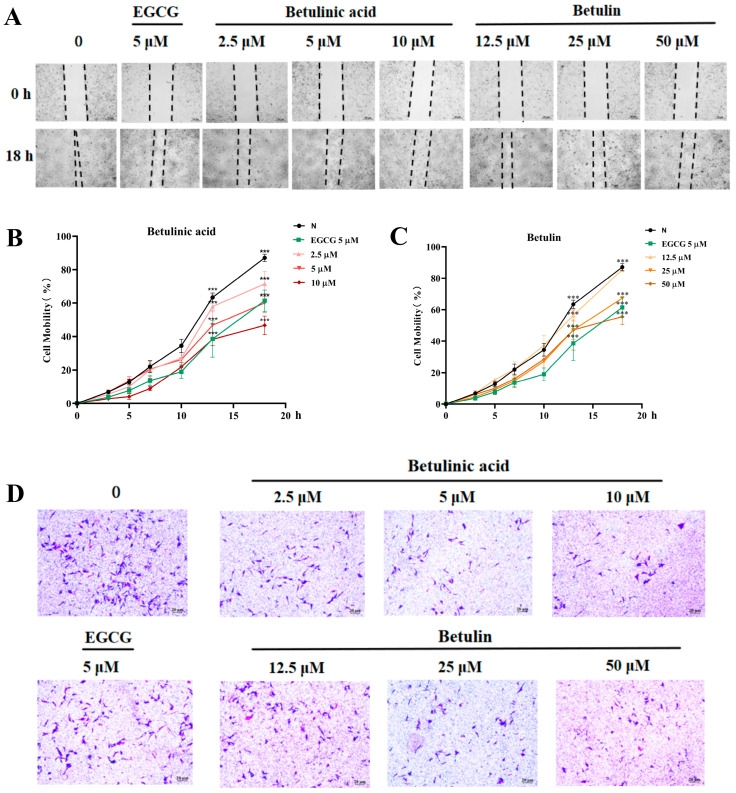
The effects of BA and BE on the migration of B16-F10 cells. (**A**–**C**) Wound closure rates measured by wound healing assay after 24 h treatment with BA and BE. Dashed lines indicate the edges of cell scratches. (**D**) Invasive capacity assessed by Transwell assay following 24 h exposure to BA and BE. Scale bar = 20 µm. Compared with the normal control group, *** *p* < 0.001 (*n* = 3).

To assess B16-F10 cell migration in response to BA and BE, both wound healing and Transwell invasion assays were employed. In the wound healing assay, BA and BE treatment significantly reduced the horizontal migratory capacity of B16-F10 cells compared with the untreated control group (Figure 3A–C). At 18 h, when the scratch in the control group was almost completely closed, the relative migration rates were 71.48%, 60.63%, and 46.65% in the BA-treated groups, and for 85.53%, 67.33%, and 55.61% in the BE-treated groups. The EGCG group showed a relative migration rate of 61.45%. Notably, BA exhibited a stronger inhibitory effect on B16-F10 cell migration than BE. Consistently, the Transwell invasion assays further confirmed that both BA and BE significantly suppressed the vertical invasion ability of B16-F10 cells compared to the control group (Figure 3D).

### 2.4. BA and BE Promote the Apoptosis of B16-F10 Cells

**Figure 4:**
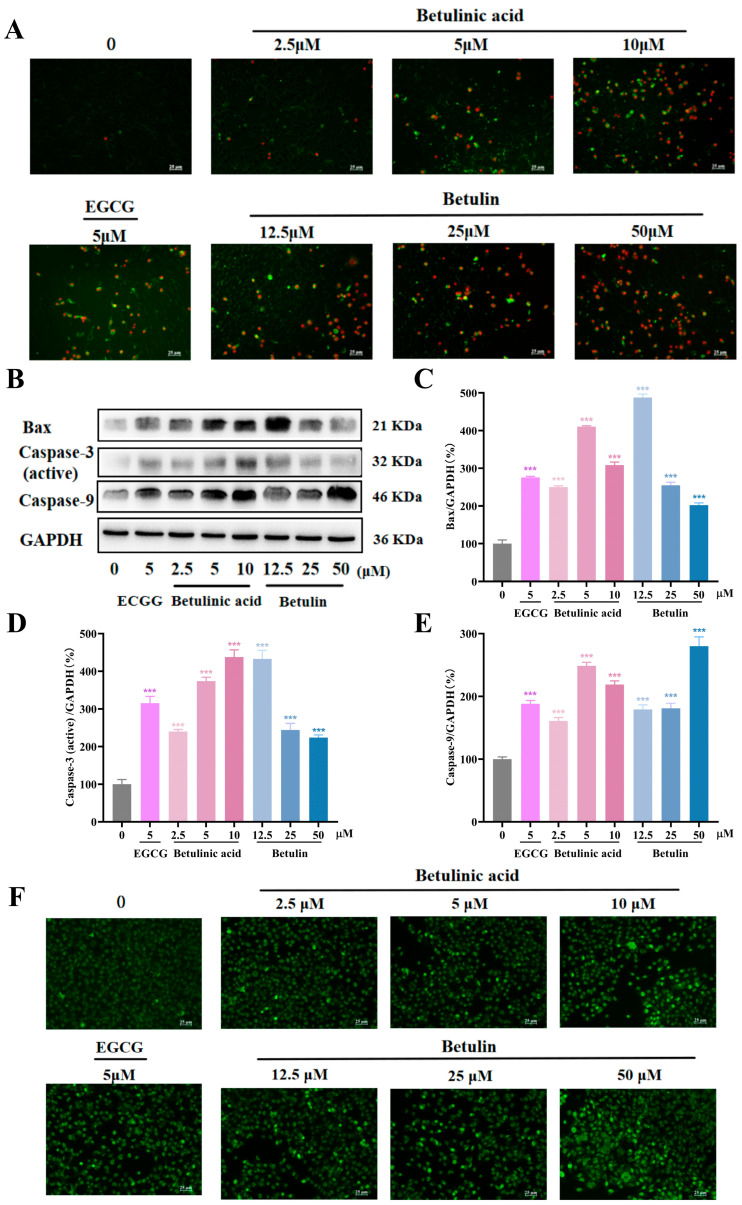
Effects of BA and BE on apoptosis and DNA damage in B16-F10 cells. (**A**) Fluorescence intensity indicating apoptosis measured with a dual-staining kit following 24 h exposure to BA and BE. Green indicates apoptosis, while red indicates necrosis. Scale bar = 25 µm. (**B**–**E**) Levels of apoptosis-related proteins determined by Western blotting following 24 h exposure to BA and BE. (**F**) Fluorescence intensity of DNA staining. Green indicates DNA damage. Scale bar = 25 µm. Compared with the normal control group, *** *p* < 0.001 (*n* = 3).

The effects of BA and BE on apoptosis in B16-F10 cells were evaluated using Annexin V-FITC/PI staining. As shown in Figure 4A, treatment with either BA or BE increased both apoptosis and necrosis levels. Consistently, Hoechst 33342 staining revealed enhanced apoptotic signals following drug treatment (Figure S2). Furthermore, we analyzed the expression of apoptosis-related proteins, including Bax, caspase-3, and caspase-9. As shown in Figure 4B–E, the protein expression levels of Bax, caspase-3, and caspase-9 were elevated relative to those in the normal control group. Additionally, DNA damage was assessed by measuring γ-H2AX levels via immunofluorescence assays (Figure 4F). The results demonstrated BA and BE enhanced γ-H2AX fluorescence intensity, indicating increased DNA damage in B16-F10 cells. Collectively, these findings suggest that BA and BE promote both DNA damage and apoptosis in B16-F10 cells.

### 2.5. Effects of BA and BE on Autophagy in B16-F10 Cells

**Figure 5:**
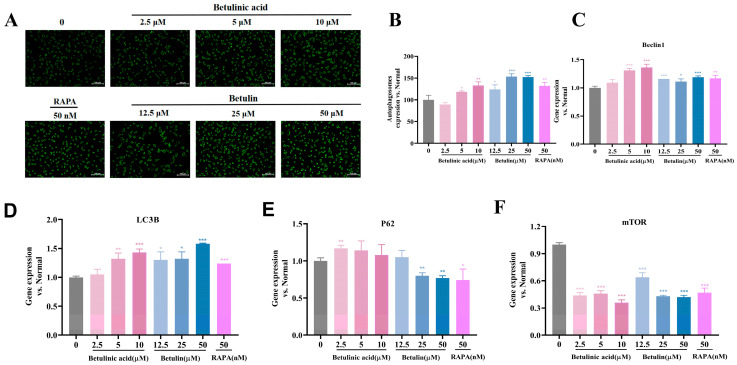
Effects of BA and BE on autophagy in B16-F10 cells. (**A**,**B**) After 24 h of treatment with BA and BE, autophagosomes fluorescence intensity was measured using staining kit and microplate reader. Green indicates autophagy. Scale bar = 100 µm. (**C**–**F**) The expression levels of autophagy marker genes were analyzed by qPCR. Compared with the normal control group, * *p* < 0.05, ** *p* < 0.01, *** *p* < 0.001, *n* = 3.

Based on network pharmacology predictions, we subsequently assessed whether BA and BE modulate autophagy in B16-F10 cells. Rapamycin (RAPA), a well-established activator, was used as a positive control in the subsequent experiments (Figure S3). As shown in Figure 5A,B, treatment with BA and BE induced a dose-dependent increase in autophagosome formation. To further characterize the autophagic response, we quantified the expression of key autophagy-related markers, including Beclin-1, LC3B, p62, and mTOR. As shown in Figure 5C–F both BE and BA upregulated Beclin-1 and LC3B mRNA expression levels, while downregulating p62 and mTOR expression levels. These findings suggested that BE and BA may suppress melanoma progression by stimulating autophagy.

### 2.6. The Regulation of BA and BE on Mitochondrial Autophagy in B16-F10 Cells

**Figure 6:**
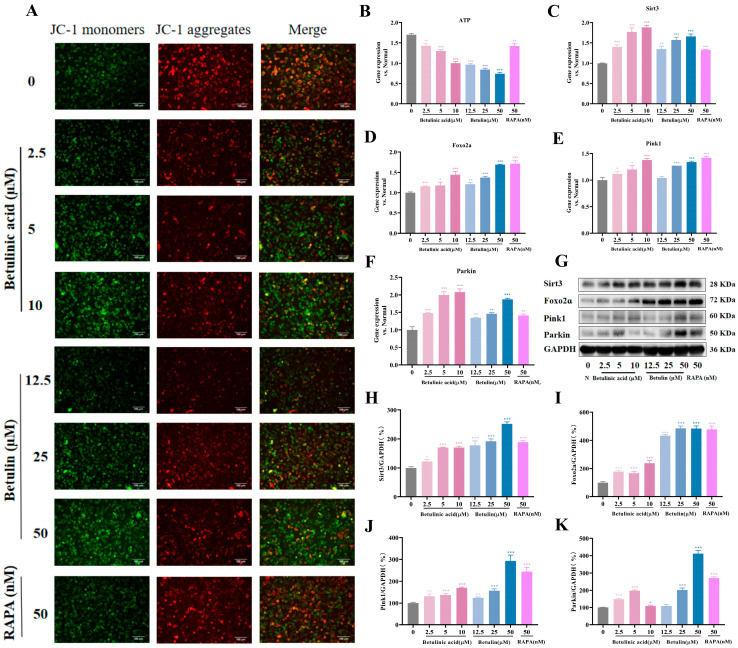
Effects of BA and BE on mitochondrial autophagy in B16-F10 cells. (**A**) Changes in mitochondrial membrane potential were measured by JC-1. Green represents JC-1 as a monomer, indicating low mitochondrial membrane potential; red represents JC-1 polymers, indicating high mitochondrial membrane potential. Scale bar = 100 µm. (**B**) ATP levels were measured after 24 h treatment with BA and BE. (**C**–**F**) Mitophagy-related pathway mRNA levels were detected via qPCR. (**G**–**K**) Mitophagy-related pathway mRNA levels were detected via Western blot. Compared with the normal cell group, * *p* < 0.05, ** *p* < 0.01, *** *p* < 0.001, *n* = 3.

To further investigate whether BA and BE inhibit melanoma progression by inducing mitochondrial stress responses, we detected cellular mitochondrial membrane potential (ΔΨm) using the JC-1 fluorescent probe. After the treatment of BA and BE, the green fluorescence increased, indicating a decrease in ΔΨm and the occurrence of apoptosis in B16-F10 cells (Figure 6A). A significant decrease in ATP synthesis efficiency often serves as a biomarker for impaired mitochondrial energy metabolism. Consistently, we observed reduced ATP levels following treatment with BA and BE (Figure 6B). These findings suggested that BA and BE induced autophagy may be associated with mitochondria. Then, we examined the Sirt3 signaling axis, which is know to regulate the initiation and efficient execution of mitophagy by maintaining ΔΨm and enhancing the stability and activity of the Pink1/Parkin proteins [27]. As shown in Figure 6C–K, BA and BE upregulated both the mRNA and protein levels of Sirt3, Foxo2a, Pink1, and Parkin. Collectively, these results indicate that BE and BA may suppress melanoma development by activating mitochondrial stress responses.

### 2.7. Effects on Autophagy-Related Pathways Expression in B16-F10 Cells

**Figure 7:**
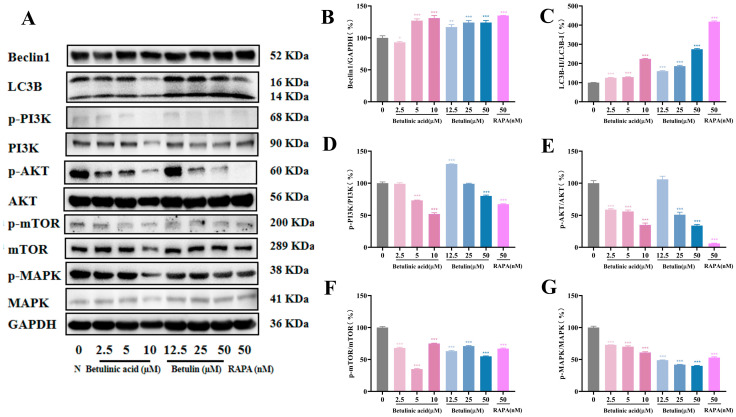
Impacts of BA and BE on autophagy-associated pathways expression in B16-F10 cells (**A**) After 24 h treatment with BA and BE, autophagy-related protein levels were assessed via Western blotting. The determination of the quantity of (**B**) Beclin1, (**C**) LC3B, (**D**) p-PI3K, (**E**) p-AKT, (**F**) p-mTOR, and (**G**) p-MAPK was normalized to GAPDH. Compared with the Normal cell group, * *p* < 0.05, ** *p* < 0.01, *** *p* < 0.001, *n* = 3.

Western blot analysis revealed that, in the BA and BE treatment groups, the protein expression of autophagy markers LC3B and Beclin1 increased significantly in a concentration-dependent manner (Figure 7A–C). Furthermore, on the basis of the outcomes derived from network pharmacological analysis, we performed in-depth exploration of the MAPK and PI3K/AKT/mTOR signaling pathways (Figure 7D–G). Following treatment with BA and BE, the expression levels of p-MAPK, p-PI3K, p-AKT, and p-mTOR exhibited a dose-dependent decrease, suggesting that regulation of autophagy-related pathways may represent a potential mechanism underlying the anti-melanoma effects of BA and BE.

### 2.8. Effects of Conditioned Culture Supernatants on HUVEC Cell Angiogenic Ability

**Figure 8:**
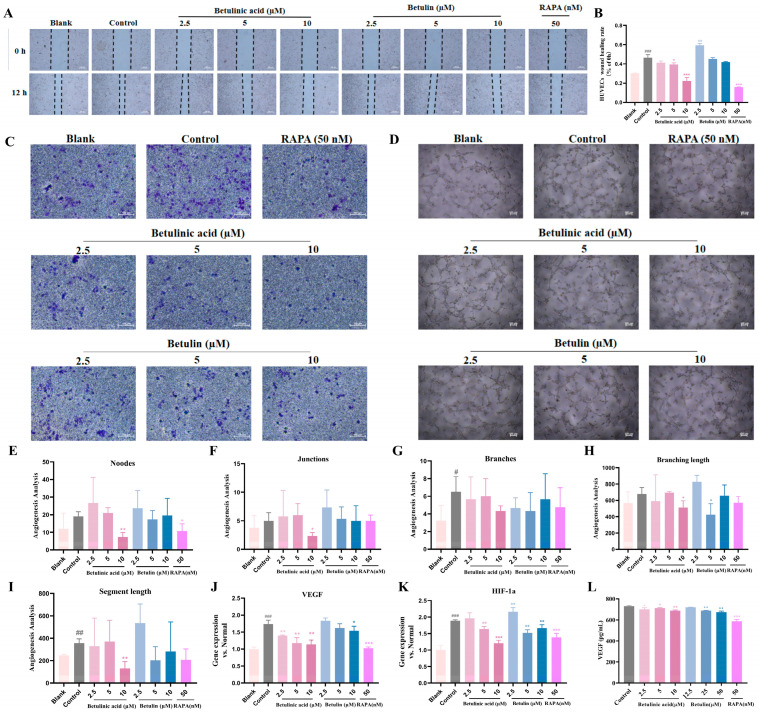
Effects of conditioned culture supernatants on HUVEC cell angiogenic ability. Conditioned media (the supernatants of B16 cells treated with BA and BE for 24 h) at different concentrations were added to HUVECs. (**A**) Wound healing assay. Dashed lines indicate Dashed lines indicate the edges of cell scratches. (**B**) Cell migration rate statistics. Scale bar = 100 µm. (**C**) Transwell invasion assays. (**D**) Lumen formation ability. Scale bar = 100 µm. (**E**–**I**) Analysis of nodes, junctions, branches, branch lengths and segment length. (**J**,**K**) The mRNA levels of VEGF and HIF-1α in HUVECs were measured by qPCR. (**L**) The concentration of VEGF in the conditioned medium was detected by Elisa. Compared with the blank group (Normal culture medium), ^#^ *p* < 0.05, ^##^ *p* < 0.01, ^###^ *p* < 0.001; Compared with the control group, * *p* < 0.05, ** *p* < 0.01, *** *p* < 0.001, *n* = 3.

Tumor cells and vascular endothelial cells are the primary cellular components of the tumor microenvironment, and their interactions persist throughout tumor progression [28,29]. To validate the anti-angiogenic activity of BA and BE, we established a co-culture system to investigate the indirect effects of conditioned medium derived from BA- or BE-treated B16-F10 cells on HUVEC cells. First, we investigated the cytotoxicity of BA and BE on HUVECs (Figure S4). We then collected the supernatants from B16 cells treated with BA or BE for 24 h, and applied conditioned media at different concentrations to HUVEC cultures. Wound healing assays demonstrated that conditioned media from BA- or BE-treated cells suppressed the horizontal migration of HUVECs in a dose-dependent manner (Figure 8A,B). Consistently, Transwell migration assays demonstrated that HUVECs exposed to conditioned media from BA- or BE-treated groups exhibited a significant, dose-dependent decrease in vertical migration capacity (Figure 8C). Moreover, BA and BE inhibited vascular nodes, junctions, branches, branch lengths and segment length, thereby suppressing angiogenesis (Figure 8D–I). These results indicated that BA and BE inhibited both the horizontal and vertical migration capabilities, as well as angiogenesis, in HUVECs. Given that HIF-1α, a key upstream inducer of VEGF, plays a crucial role in tumor angiogenesis [30], we quantified VEGF and HIF-1α expression in HUVECs by qPCR. Both VEGF and HIF-1α mRNA levels were increased in HUVECs exposed to culture supernatant from untreated B16 cells, whereas conditioned media from BA- and BE-treated media suppressed the expression of these mRNAs (Figure 8J,K). It was further confirmed by Elisa experiments that the concentration of VEGF in the supernatant of B16-F10 cells was lower compared with the control group (Figure 8L).

### 2.9. In Vivo Animal Study Evaluation of BA’s Anti-Melanoma Effects

**Figure 9:**
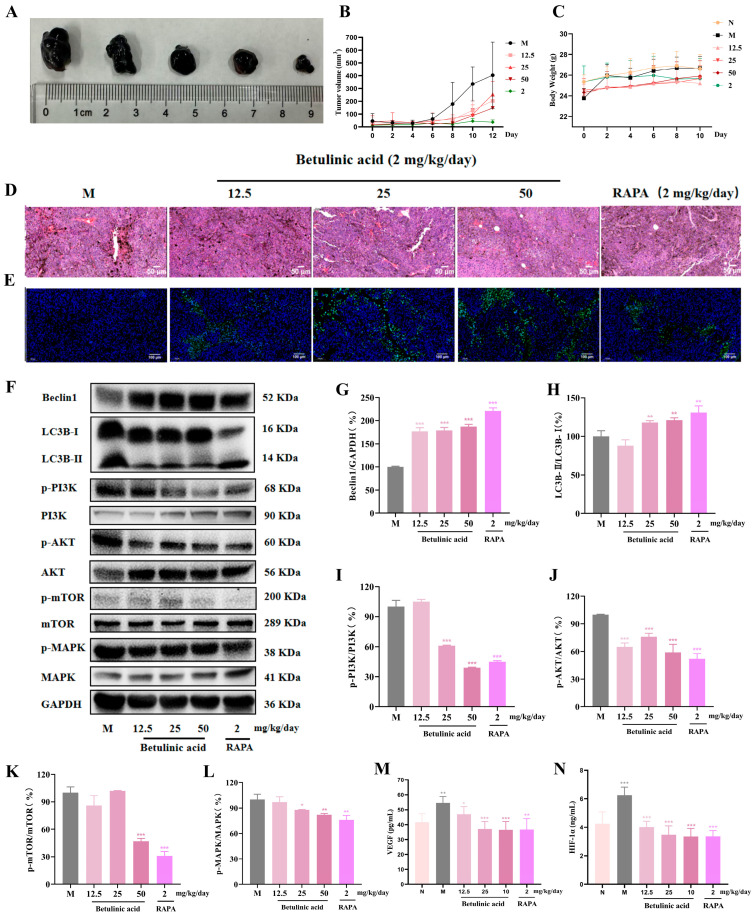
In vivo animal study evaluation of BA’s anti-melanoma effects. (**A**,**B**) Inhibitory effect of BA on tumor volume. (**C**) The changes in the body weight of mice. (**D**) H&E staining. (**E**) TUNEL fluorescence staining (blue indicates nuclei, green represents apoptotic cells). (**F**) Expression levels of autophagy-related proteins in mouse tumor tissues following BA treatment. The quantification of (**G**) Beclin1, (**H**) LC3B, (**I**) p-PI3K, (**J**) p-AKT, (**K**) p-mTOR, and (**L**) p-MAPK was normalized to GAPDH and presented as the ratio (percentage) relative to the model group. (**M**,**N**) The concentrations of VEGF and HIF-1α in serum were detected by Elisa. Compared with the model group, * *p* < 0.05, ** *p* < 0.01, *** *p* < 0.001, *n* = 3.

In vitro cellular assays revealed that BA a stronger ability to promote apoptosis of melanoma cells and inhibit angiogenesis than that of BE. Additionally, the PI3K/AKT signaling pathway is more significantly modulated by BA. Based on these compelling in vitro results, subsequent animal experiments employed BA exclusively as the interventional agent to further validate its biological activity in vivo. Phenotypically, BA significantly inhibited melanoma tumor growth in mice (Figure 9A,B). As shown in Figure 9C and Figure S5, compared with the normal group, no statistically notable discrepancies were detected in body weight and the organ indicators of BA-treated mice (*p* > 0.05). H&E and TUNEL staining revealed that, in the BA treatment group, lymphocyte infiltration was reduced, fewer new capillaries were present peripherally, melanin secretion decreased, and apoptosis increased (Figure 9D,E and Figure S6A,B). Similarly, following BA treatment, there was a dose-dependent increase in Beclin1 and LC3B protein levels (Figure 9G,H), accompanied by decreased levels of p-MAPK, p-PI3K, p-mTOR, and p-AKT proteins (Figure 9I–L). As shown in Figure 9M,N, compared to normal mice, model group mice exhibited higher serum concentrations of VEGF and HIF-1α. BA treatment dose-dependently reduced these levels. These results further validated that modulation of autophagy-related pathways may represent a potential mechanism underlying the anti-melanoma effects of BA.

## 3. Discussion

Melanoma is one of the most invasive and deadly forms of skin cancer. Its danger lies not only in the rapid malignant transformation originating from mela-nocytes but also in its inherent traits of early invasion and rapid metastasis [11]. Natural compounds have attracted increasing attention as therapeutic candidates for human diseases because of their relatively low toxicity and potent bioactivity. Betulinic Acid (BA) and betulin (BE), which are particularly abundant in the bark of *Betula platyphylla* Suk., have demonstrated pronounced anti-tumor activities [31,32]. Previous studies have shown that BA can suppress cancer cell proliferation without causing adverse effects on normal cells [33]. Moreover, BA inhibits tumorigenesis in various cancers via apoptotic, autophagic [34] and metastasis-related pathways [35]. Xu et al. showed that BA induced apoptosis in human cervical cancer cells by downregulating PI3K/AKT signaling [36]. Other studies have shown that BA exerts anti-angiogenic effects by blocking the interaction between HIF-1α and the VEGF promoter [37]. Zehra et al. discovered that betulin isolated from *Quercus incana* exhibited apoptotic and anti-metastatic activities against non-small cell lung cancer cells [38]. Consistent with these findings, our results confirmed that BA and BE significantly promoted apoptosis in B16-F10 cells and effectively inhibit the angiogenic ability of HUVEC cells. Additionally, we found that BA and BE regulated mitochondrial autophagy in tumor cells. Nonetheless, the molecular mechanisms underlying BA- and BE-mediated anti-melanoma effects are still undefined. Therefore, this study investigated whether BA and BE mediate autophagy through the PI3K/AKT/mTOR and MAPK signaling pathways, induce apoptosis, and thereby exert anti-melanoma effects.

Autophagy plays a crucial role in various diseases, particularly in cancer. Our results indicated that BA and BE promoted the generation of autophagosomes, increased the expression of Beclin1 and LC3B, and downregulated the mRNA levels of mTOR and P62. We hypothesized that BA and BE may affected melanoma by activating autophagy. Previous studies have reported that many conventional anticancer drugs induce cell death by causing mitochondrial damage [39]. Sirt3 has been reported to exert both tumor-promoting and tumor-suppressive effects in cancer [40]. SIRT3 could promote the deacetylation process and nuclear translocation of FOXO3a to activate Parkin protein, thereby effectively regulating mitochondrial autophagy [41]. In this study, BA and BE decreased the ΔΨm and simultaneously reduced ATP production, leading to impaired mitochondrial energy metabolism. Subsequently, we examined the Sirt3/FOXO3a signaling pathway and observed that Sirt3 was activated, with significantly upregulated expression of FOXO3a, PINK1, and Parkin proteins, thereby inducing enhanced cellular autophagy. Based on these, we speculated that BA and BE may affect melanoma by activating mitophagy. To further investigate whether BA induces mitophagy by mediating the SIRT3/FOXO3/PINK1/Parkin pathway, future studies will evaluate Parkin translocation or the loss of mitochondrial proteins (TOM20/COXIV) in a manner that is prevented by lysosomal inhibitors.

Network pharmacology is a powerful tool to generate integrated drug-component-gene-disease networks, enabling the rapid identification of active components and therapeutic targets of traditional Chinese medicine (TCM) [42]. Network pharmacology analysis revealed that the antitumor effects of BA and BE are closely associated with their regulation of apoptosis, cell migration, angiogenesis, and autophagy. These effects are primarily mediated via the PI3K/AKT and MAPK signaling pathways. Melanoma exhibits a high proliferative capacity largely due to frequent driver mutations, such as BRAF and NRAS. These mutations persistently activate pro-proliferative pathways, including PI3K/AKT/mTOR and MAPK, disrupt cell cycle regulation, and disturb the “proliferation–apoptosis” balance in melanocytes, ultimately leading to uncontrolled rapid proliferation [6]. Western blot analyses in both in vitro and in vivo demonstrated that BA treatment increased expression of Beclin1 and LC3B proteins, while significantly decreasing expression of PI3K, AKT, mTOR, and MAPK proteins in murine melanoma cells. These findings may correlate with activated autophagy in mouse melanoma, as evidenced by increased autophagosome formation, reduced melanin content, and enhanced apoptotic cell death. Mechanistically, activation of the PI3K-AKT pathway negatively regulates autophagy through AKT-mediated mTORC1 activation, which suppresses autophagosome biogenesis. Inhibiting this cascade relieves mTOR suppression, thereby promoting autophagy and apoptosis in melanoma cells. Concurrently, aberrant activation of the MAPK signaling pathway inhibits autophagy. The observed therapeutic effects likely result from multitarget engagement within KEGG pathways-specifically targeting key nodes such as PI3K-AKT and MAPK-highlighting a potential mechanism for synergistic multipathway modulation in melanoma prevention and treatment. However, the specific functional differences between autophagy activation and autophagy inhibition remain to be further elucidated. Subsequent studies will utilize lysosomal inhibitor intervention experiments to determine whether this regulatory process induces mitochondrial stress by mediating the SIRT3/FOXO3/PINK1/Parkin pathway. Additionally, by activating the AKT/mTOR or MEK/ERK pathways, we aim to clarify the intrinsic relationships between these signaling pathways and the processes of apoptosis and autophagy.

Angiogenesis supplies nutrients to melanoma, promoting tumor growth and metastasis. Consistent with this, conditioned medium from BA- and BE-treated melanoma cells inhibited the migration, invasion, and tube formation capabilities of HUVECs. We hypothesized that this effect resulted from the drugs suppressing the production of angiogenic growth factors by HUVECs. Our qPCR results showed that supernatants from the drug-treated groups significantly downregulated VEGFA and HIF-1α expression, thereby exacerbating the deficiency of oxygen and nutrients within the melanoma. However, subsequent studies will control for conditioned medium vehicle, pH and osmotic pressure, and compare direct vs. indirect effects (e.g., BA/BE in fresh medium) to further investigate the influence of additional factors on angiogenesis-related experimental outcomes. These findings confirm that BA and BE inhibit angiogenesis in melanoma. C57BL/6 male mice effectively model the initiation, growth, and metastasis of human melanoma. This allows for efficient investigation of tumor mechanisms and the screening of drug and immunotherapy regimens. Furthermore, tumor mutation characteristics and immune pathways are conserved between mice and humans, research findings offer reliable references for the clinical translation of human melanoma studies and help address scientific challenges associated with the disease. This study preliminarily investigated the effects of BA and BE on the migration of B16-F10 cells; however, further research using human melanoma cells and a mouse melanoma metastasis model is necessary.

In conclusion, this study indicated that BA and BE promoted B16-F10 cells apoptosis and autophagy, suppressed HUVEC angiogenesis, and modulated the PI3K/AKT/mTOR and MAPK signaling pathways to exert anti-melanoma effects. In vitro and in vivo experiments have demonstrated that BA has the potential to become a candidate for anti-melanoma applications.

## 4. Materials and Methods

### 4.1. Materials

B16-F10 cells were provided by the Shanghai Cell Bank of the Chinese Academy of Sciences. HUVEC cells were provided by the School of Life Science and Health Engineering of Jiangnan University. Betulinic acid (BA) and Betulin (BE) were provided by MedChemExp (MCE, Monmouth Junction, NJ, USA). Epigallocatechin-3-gallate (EGCG) was purchased from Nanjing Plant Origin Biological (Nanjing, China). Rapamycin (RAPA) was provided by Shanghai Titan Scientific Co., Ltd. (Shanghai, China). Penicillin, streptomycin, trypsin digestion solution and fetal bovine serum (FBS) were obtained from Gibco-Invitrogen (Thermo Fisher Scientific, Waltham, MA, USA). Antibodies (Bax, Caspase-3(active), Caspase-9, PI3K, p-PI3K, Beclin1, LC3B, Sirt3, Foxo2a, Pink1, Parkin, AKT, p-AKT, MAPK p-MAPK, mTOR and p-mTOR).

### 4.2. Network Pharmacology Analysis

#### 4.2.1. Target Screening

The SMILES of the BA and BE were retrieved from PubChem database (https://pubchem.ncbi.nlm.nih.gov, accessed on 2 February 2025), and uploaded to Swiss Target Prediction (http://www.swisstargetprediction.ch, accessed on 2 February 2025), with Homo sapiens selected as the target species to predict potential targets of BA and BE. Potential targets with zero confidence in the prediction results were excluded. Then, using the UniProt database (https://www.uniprot.org/, accessed on 2 February 2025) and STRING 12.0 (https://cn.string-db.org/, accessed on 2 February 2025), gene symbol names corresponding to drug targets were normalized.

#### 4.2.2. Screening for Potential Therapeutic Targets

To identify relevant targets, the keyword “Melanoma” was used in the GeneCards (https://www.genecards.org/, accessed on 2 February 2025) and DrugBank (https://go.drugbank.com/, accessed on 2 February 2025) databases. The disease genes were intersected with the target genes corresponding to BA and BE using jvenn online platform (https://www.bioinformatics.com.cn/static/others/jvenn/example.html, accessed on 2 February 2025). The overlapping genes were defined as candidate therapeutic targets for BA/BE against melanoma.

#### 4.2.3. Construction of Protein–Protein Interaction Network

The overlapping target were imported into the STRING online data analysis platform to construct a PPI network, with Homo sapiens selected and a high confidence score threshold of 0.90 applied. Proteins without interactions were identified, and a hidden network of these isolated proteins was generated. The acquired protein interaction data was imported into Cytoscape in “.tsv” format for the topological analysis of the protein–protein interaction network using Cytoscape 3.9.1 (https://www.cytoscape.org/, accessed on 2 February 2025). Targets with low interaction degrees were removed. The remaining targets were ranked based on their degree values, and the top-ranked targets were selected for network visualization.

#### 4.2.4. Gene Ontology (GO) and Kyoto Encyclopedia of Genes and Genomes (KEGG) Enrichment Analysis

The DAVID database (https://davidbioinformatics.nih.gov/, accessed on 2 February 2025) was used to perform GO analyses and KEGG pathway for the intersection of BA/BE with Melanoma targets. The top 20 terms/pathways with the highest gene counts were exported and visualized using the Weishengxin online platform (https://www.bioinformatics.com.cn, accessed on 2 February 2025).

### 4.3. Determination of Free Radical Scavenging Ability of DPPH

The antioxidant capacity was analyzed by measuring the scavenging ability of DPPH (300267, Sigma-Aldrich, St. Louis, MO, USA) free radicals. 100 µg/mL DPPH stock solution was made by dissolving the powder in methanol. Further dilutions were made with methanol for BA/BE (final concentrations: 1, 5, 10, 15, 20, 25, 30, and 35 mM) and EGCG (final concentrations: 5, 10, 25, 50, 100, 200, and 300 µM). In a 96-well plate (Suzhou CellPro Biotechnology Co., Ltd. Suzhou, China), equal volumes (1:1) of the DPPH solution and each test compound were mixed and incubated at 37 °C in the dark for 30 min. Vitamin C was used as the positive control. Triplicate wells were prepared for each sample. A microplate reader (BioTek Instruments, Inc., Winooski, VT, USA) was employed to determine the absorbance at 517 nm. The equation is as follows:

DPPH radical scavenging activity (%) = [1 − (A_1_ − A_2_)/A_0_] × 100

where A_1_ is the absorbance of the sample, A_2_ is the absorbance of the DPPH solution, and A_0_ is the absorbance of the ethanol.

### 4.4. Cell Counting Kit-8 (CCK-8) Assay

The Cell Counting Kit-8 (CCK-8, MA0218, Meilun Dalian, China) was utilized to evaluate the effects of samples on cell proliferation. Briefly, each well of the 96-well plate was inoculated with 1.8 × 10^4^ cells and incubate at 37 °C in an incubator with 5% CO_2_ for 24 h to reach 80% confluency. After an additional 24 h of drug treatment, a 10% CCK-8 solution was added, and the cells were incubated under the same conditions. Finally, a microplate reader was employed to determine the absorbance at 450 nm.

### 4.5. Colony Formation Assay

Each well of the 12-well plate was inoculated with 5000 B16-F10 cells. Following treatment with BA, BE, and EGCG for 24 h, the cells were maintained in complete DMEM medium for 5 to 7 days to promote colony formation. The anti-proliferate activity was assessed by evaluating the differences in the number and size of colonies.

### 4.6. Wound Healing Assay

The ibidi Culture-Insert 2 Well (80209, ibidi, Gräfelfing, Germany) cell migration plugin inserts were placed into a 24-well plate, and B16-F10 cells at a density of 3.5 × 10^4^ cells per well were seeded into the two-well inserts. The cells were cultured in an incubator until a confluent cell layer formed. After the cells reached 100% confluency, the inserts were removed, and the wells were washed twice with PBS. BA (2.5, 5, 10 μM), BE (12.5, 25, 50 μM), and EGCG (5 μM) were applied for 24 h. The cells were then incubated in a culture incubator and observed for cell migration every 4 h. Photos of the scratches were taken, and the scratch area was analyzed using ImageJ 1.46r/Java 1.6.0 software for calculation.

### 4.7. Transwell Assays

A Transwell chamber assay (Corning Inc., Corning, NY, USA) was used to investigate cell migration. The 3 × 10^4^ B16-F10 cells suspension was placed in the upper chamber, while the medicine was added to the lower chamber. The cells were incubated for 56 h, then fixed with a 4% paraformaldehyde solution and stained with a 0.1% crystal violet solution for 10 min. After washing three times with PBS, images were captured using a microscope. ImageJ software was used to analyze the images.

### 4.8. Apoptosis Measured by Annexin V-FITC/PI Staining

To further explore apoptosis, Annexin V-FITC Kit (E606336-0100, Sangon Biotech, Shanghai, China) was used for testing in accordance with the manufacturer’s instructions. After treatment with betulin (12.5, 25, 50 μM), betulinic acid (2.5, 5, 10 μM), and EGCG (5 μM) for 24 h, the cells were stained with Annexin V-FITC and PI staining solution. The cells were incubated on ice in a light-protected environment for 20 min before being photographed using a fluorescence microscope (TS2-S-SM, Nikon, Japan).

### 4.9. γ-H2AX Immunofluorescence Method

The extent of drug-induced DNA damage in cells was assessed using a DNA damage detection kit (C2035S, Beyotime Biotech. Inc., Shanghai, China). Briefly, cells were fixed with a fixative solution for 10 min and washed three times with washing buffer. Subsequently, they were blocked with immunostaining blocking buffer at room temperature for 20 min. A rabbit anti-γ-H2AX monoclonal antibody was added and incubated for 1 h, then wash three times with washing buffer. Then, an anti-rabbit Alexa Fluor 488 secondary antibody (Proteintech Group Inc., Rosemont, IL, USA) was applied under light protection at room temperature for 1 h, followed by another three washes. Stain at room temperature in the dark with nuclear staining solution for 5 min, and wash with washing solution three times. Finally, an anti-fade mounting medium was added to each well, and the intensity of the green fluorescence signal from γ-H2AX foci was visualized under a fluorescence microscope.

### 4.10. Detection of Mitochondrial Membrane Potential

Early apoptosis in cells was detected using a JC-1 staining buffer kit (C2006, Beyotime Biotech. Inc., Shanghai, China). Briefly, during the logarithmic growth phase, each well of the 12-well plate was inoculated with 2.5 × 10^5^ B16-F10 cells. Once the cell density reaches 80%, drug treatment should be administered for 24 h. The culture medium was removed, and the cells were incubated with 1 mL of fresh medium containing the JC-1 working solution at 37 °C under 5% CO_2_ for 20 min. Subsequently, the supernatant was aspirated, and the cells were washed twice with 1× buffer. Finally, fluorescent images were acquired using a fluorescence microscope.

### 4.11. Detection of ATP Content

An enhanced ATP assay kit (Beyotime Biotech. Inc., Shanghai, China) was employed to determine the cellular ATP content. Briefly, during the logarithmic growth phase, each well of the 12-well plate was inoculated with 2.5 × 10^5^ B16-F10 cells. Once the cell density reaches 80%, drug treatment should be administered for 24 h. cells were lysed in lysis buffer and centrifuged to collect the supernatant as samples. The samples were then diluted 10-fold with lysis buffer. In a black 96-well plate, 100 µL of ATP detection reagent was added to each well, followed by the addition of 10 µL of the diluted samples and serially diluted ATP standard solutions. Luminescence signals (RLU values) were measured using a chemiluminescence microplate reader, and ATP concentrations were calculated based on the standard curve.

### 4.12. Detection of Autophagy Level

According to the manufacturer’s product insert, autophagy level was detected by monodansylcadaverine (MDC, Beyotime Institute of Biotechnology, Beijing, China). Briefly, during the logarithmic growth phase, each well of the 12 and 96 well plate was inoculated with 2.5 × 10^5^ and 1.8 × 10^4^ B16-F10 cells. Once the cell density reaches 80%, drug treatment should be administered for 24 h. B16-F10 cells were incubated with MDC (1×) for 60 min in the dark. After washing with Assay Buffer (1×) three times, HDPC cells were imaged under fluorescence microscopy (TS2-S-SM, Nikon, Tokyo, Japan), and the absorbance was determined at an excitation wavelength of approximately 335 nm and an emission wavelength of approximately 512 nm using a multimode microplate reader (BioTek Instruments, Inc., Winooski, VT, USA).

### 4.13. Evaluation of HUVEC Cell Lumen Formation Ability

On the day before the cell experiment, the Matrigel (354234, Corning Inc., Corning, NY, USA) was subjected to overnight thawing at 4 °C. Pre-cooling of the 96-well plate and pipette tips was carried out beforehand. 50 μL per well Matrix adhesive was added to the precooled 96 well plate (operated on ice). The plate was incubated for 45 min at 37 °C in a 5% CO_2_ atmosphere to facilitate gelation. HUVECs were then plated onto the Matrigel matrix at a seeding density of 1.8 × 10^4^ cells per well. After 4h treatment, lumen formation in HUVEC cells was observed under a microscope and analyzed using ImageJ software.

### 4.14. Quantitative Real-Time PCR (qPCR) Assay

Extract RNA from cells using pre-chilled Trizol (R0016, Beyotime Biotech. Inc., Shanghai, China), and store the extracted RNA in DEPC-treated Water. Subsequently, determine the RNA concentration using a NanoDrop spectrophotometer (Thermo Fisher Scientific Inc., Waltham, MA, USA). Use 400 ng of total RNA as a template to synthesize cDNA according to the protocol provided by the First Strand cDNA Synthesis Kit (D7168, Beyotime Biotech. Inc., Shanghai, China). Using cDNA as a template, add SYBR (D7260, Beyotime Biotech. Inc., Shanghai, China) and primers (Table 1), and perform amplification according to the protocol provided by the reagent manufacturer on the CFX96 Touch Real-Time PCR System (Bio-Rad, Hercules, CA, USA). Each sample should be run in at least three parallel wells to reduce errors in data analysis.

### 4.15. Western Blot Assay

The Bradford protein assay kit (P0006, Beyotime Biotech. Inc., Shanghai, China) was used to quantify proteins. Each well had a volume of 10 µL and contained 30 µg of protein. After SDS-PAGE, proteins were blotted onto a 0.45 µm PVDF membrane. The membrane was blocked with 5% BSA solution, washed three times, and then incubated with the primary antibody at 4 °C overnight. Secondary antibody (1:10,000 dilution) was applied to the membrane and incubated for 1 h. Development was performed using the Tanon gel imaging system (Tanon Life Science Co., Ltd., Shanghai, China). Image J software was used for grayscale analysis.

### 4.16. Melanoma Mouse Modeling

Male C57BL/6J mice, aged 7 weeks and weighing 18–23 g, were purchased from Zhejiang Vital River Laboratory Animal Technology Co., Ltd. (Zhejiang, China). The mice were housed in accordance with the guidelines of the Animal Ethics Committee of Jiangnan University (Ethics No.: JN.No20250615c0320715[356]). All animal procedures were approved by this committee.

Exponentially growing B16-F10 cells were trypsinized, collected into 50 mL tubes, centrifuged, and resuspended in PBS at a concentration of 1 × 10^6^ cells per mouse. The cells were then inoculated into the right flank region of the mice. When the average tumor volume reached 40–60 mm^3^ (n = 6 per group), treatment commenced. Mice received BA at doses of 12.5, 25, or 50 mg/kg, RAPA at 2 mg/kg, or saline (model group). Drug administration was performed daily via intraperitoneal injection. Measurements of body weight and tumor volume were taken at 2-day intervals. When the tumor length reached approximately 12 mm, the mice were euthanized using humane methods. Tumor tissues, along with lungs, kidneys, liver, spleen, and heart, were excised and weighed. Part of the tumor tissue was rapidly frozen in liquid nitrogen for use in Western blotting, while the remaining tissue was fixed in 4% paraformaldehyde for histological evaluation.

### 4.17. H&E Staining and TUNEL Assay

Portions of tumors fixed with 4% paraformaldehyde were used for histological analysis. The tumor tissues were embedded in paraffin and sectioned into 5-micrometer-thick slices. These sections were stained with Hematoxylin and Eosin (H&E). Concurrently, apoptosis levels in the sections were assessed using the TUNEL assay.

### 4.18. ELISA Assay

After collecting blood from mouse eyes, centrifuge the samples at 4 °C and 4000 rpm for 15 min, then collect the supernatant. Perform quantitative detection of vascular endothelial growth factor (VEGF, KE10220, Proteintech Group Inc., Rosemont, IL, USA) and hypoxia-inducible factor-1α (HIF-1ɑ, CSB-E08541m, Wuhan Huamei Biotech Co., Ltd., Wuhan, China). A microplate reader was employed to determine the absorbance at 450 nm.

### 4.19. Statistical Analysis

Statistical analysis was conducted via GraphPad Prism 10.3.1. All experiments were conducted with a minimum of three independent replicates, and results are expressed as the mean (standard deviation). Intergroup differences were analyzed using one-way ANOVA, with *p* < 0.05 considered statistically significant.

## Figures and Tables

**Table 1:** Primer sequences of the genes.

Primers	Forward Primer	Reverse Primer
Beclin1	ATGGAGGGGTCTAAGGCGTC	TGGGCTGTGGTAAGTAATGGA
LC3B	CGTCCTGGACAAGACCAAGTTCC	CAGGAGGAAGAAGGCTTGGTTAGC
P62	GACCCATCTACAGAGGCTGAT	GCCTTCATCCGAGAAACCCA
mTOR	TGGCATAACAGATCCTGACCCTG	GCTTGTAAGTTTTCTGCCTGGG
Sirt3	ATCATGGCGCTAAGCGGTC	TCTCCCACCTGTAACACTCC
Foxo2α	GAAGGAGCCGAGGTAGCTG	CTTGGGCTCTTGCTCTCTCC
Pink1	GAGGAGCAGACTCCCAGTTC	CCAGGGACAGCCATCTGAGT
Parkin	CCGGTGACCATGATAGTGTT	TCCTTGAGCTGCAAGATGCT
β-actin	GCAGGAGTACGATGAGTCCG	ACGCAGCTCAGTAACAGTCC
VEGFA	TGCTGTCTTGGGTGCATTGG	AGGTCTCGATTGGATGGCAG
HIF-1α	GAACGTCGAAAAGAAAAGTCTCG	CCTTATCAAGATGCGAACTCACA
GAPDH	TGACTTCAACAGCGACACCCA	CACCCTGTTGCTGTAGCCAAA

## Data Availability

The original contributions presented in this study are included in the article/Supplementary Materials. Further inquiries can be directed to the corresponding author.

## Supplementary Materials

The following supporting information can be downloaded at: https://www.mdpi.com/article/10.3390/ijms27020576/s1.

## Abbreviations

MM: Malignant melanoma; BA: Betulinic acid; BE: Betulin; PI3K: Phosphatidylinositol 3 kinase; EGCG: Epigallocatechin-3-gallate; RAPA: Rapamycin; VC: Vitamin C; AKT: Protein kinase B; Beclin-1: Bcl-2-interacting protein1; Bcl-2; B-cell lymphoma-2; MAPK: Mitogen-activated protein kinase; H&E: Hematoxylin and Eosin; DPPH: 2,2-diphenyl-1-picrylhydrazyl; CCK-8: Cell Counting Kit-8; qPCR: Quantitative Real-Time PCR; VEGF: Vascular endothelial growth factor; ROS: Reactive oxygen species; HIF-1α: Hypoxia-inducible factor-1α; MDC: Monodansylcadaverine; UV: Ultraviolet.
