# Supplementary material for: Betulinic Acid and Betulin Suppress Melanoma Growth by Modulating Apoptosis and Autophagy via PI3K/AKT/mTOR and MAPK Pathways
Source: Int J Mol Sci. 2026 Jan 6;27(2):576. doi: 10.3390/ijms27020576 (PMC12841062; doi:10.3390/ijms27020576)
Supplement: Supplementary file 1 [file ijms-27-00576-s001.zip › ijms-4020008-supplementary.pdf]

Betulinic Acid and Betulin Suppress Melanoma Growth by Modulating Apoptosis and Autophagy via PI3K/AKT/mTOR and MAPK Pathways

1. Materials and Methods

1.1 Cell Counting Kit-8 (CCK-8) assay

The Cell Counting Kit-8 (CCK-8, MA0218, Meilun Dalian, China) was utilized to evaluate the effects of samples on cell proliferation. Briefly, 1.8×104 cells were plated in each well of a 96-well plate and incubate at 37℃ in an incubator with 5% CO2 for 24 h to reach 80% confluency. After treatment with the drugs for an additional 24 h, CCK-8 solution was added according to the manufacturer's instructions and incubated under the same cinditions. Finally, optical density (OD) was measured at 450 nm.

1.2 Hoechst 33342 Staining Assay

Hoechst Staining Kit (C0003, Beyotime Biotech. Inc., Shanghai, China) was used to detect apoptosis. The operation steps were carried out in accordance with the product manual. Briefly, after 24 hours of treatment with BA and BE, the cells were washed twice with PBS, and fixed with 250 µL of 4% paraformaldehyde fixation solution for 10 min at room temperature. And then, cells were stained with 250 µL of Hoechst 33258 staining solution for 5 min at room temperature, protected from light. Finally, the fluorescence intensity of Hoechst 33258 was observed using a fluorescence microscope.

2. Result

2.1 Supplementary Material 1 To explore the effect of EGCG on the cytotoxicity of B16-F10 cells

After a 24-hour treatment with EGCG, the viability of B16-F10 cells was assessed using the CCK-8 assay. As shown in Figure S1, compared to the control

group (untreated cells), EGCG exhibited concentration-dependent inhibitory effects on B16-F10 cell survival. Since all tested concentrations maintained cell viability above 80%, it is recommended that the maximum EGCG concentration for subsequent experiments be set at 5 μM.

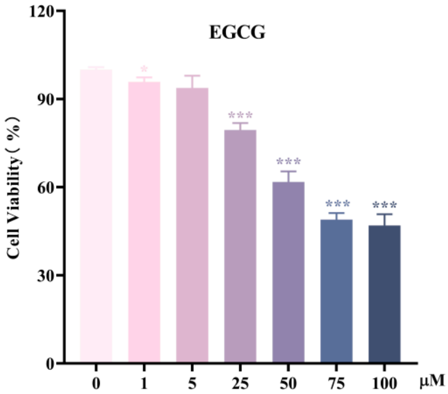

Figure S1. The effect of EGCG on the viability of B16-F10 cells was determined by the CCK-8 method. * $p < 0.05$; *** $p < 0.001$.

2.2 Supplementary Material 2 The effects of BA and BE on the apoptosis of B16-F10 cells were evaluated by Hoechst 33342 staining results

Hoechst 33342 staining results revealed increased blue fluorescence intensity, indicative of apoptotic nuclear morphology, compared to the untreated control group, demonstrating a dose-dependent response (Figure S2A,B). This finding suggest that BA and BE treatments induce apoptosis in B16-F10 cells in a dose-dependent manner.

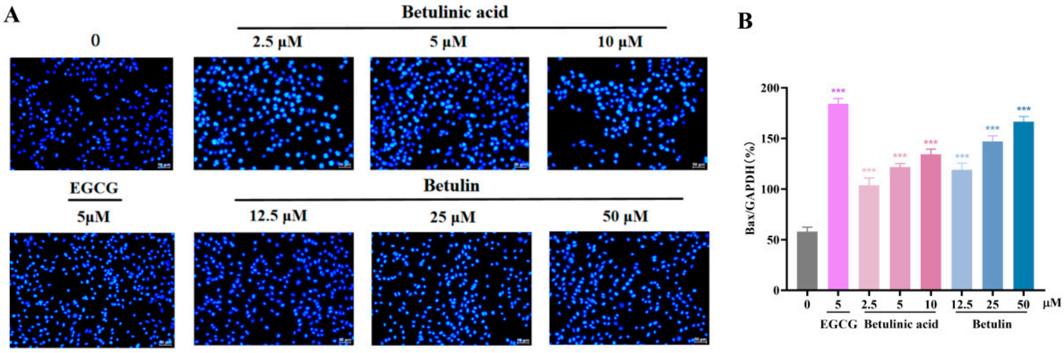

Figure S2. (**A**,**B**) Hoechst 33342 staining was used to assess the effects of BA and BE

on apoptosis in B16-F10 cells. (A) Blue indicates apoptosis. Scale bar = 50 μm. *** p < 0.001.

2.3 Supplementary Material 3 To explore the effect of RAPA on the cytotoxicity of B16-F10 cells

RAPA primarily functions as an autophagy activator and also exhibits immunosuppressive properties, therapeutic potential for autoimmune diseases, and anticancer effects. Subsequent experiments employed RAPA as a positive control. As shown in Figure S3, cell viability increased at a concentration of 100 nM, with no significant cytotoxicity observed in B16-F10 cells across the tested range of 1–50 nM. At 50 ng/mL, the cell survival rate reached 83.0% compared to untreated controls. It is recommended that the RAPA concentration be 50 nM in subsequent experiments.

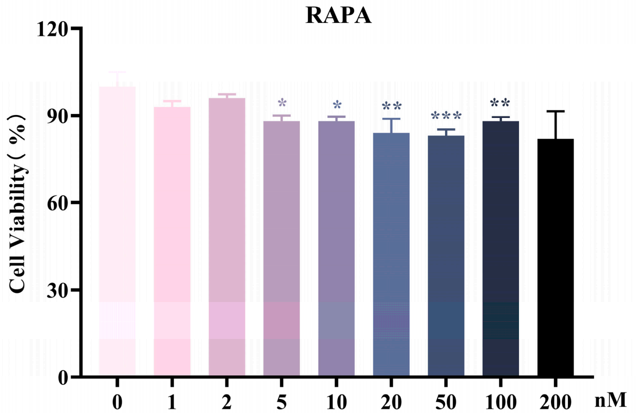

Figure S3. The effect of RAPA on the viability of B16-F10 cells was determined by the CCK-8 method. * $p < 0.05$; ** $p < 0.01$; *** $p < 0.001$

2.4 Supplementary Material 4 To explore the effect of BA, BE and RAPA on the cytotoxicity of HUVECs cells

To investigate the inhibitory effects of BA and BE on the migration and angiogenesis of HUVECs induced by the supernatant of B16-F10 cells, we first assessed the cytotoxicity of BA and BE on HUVECs. Considering that the cell inhibition rate exceeded 80% and aligned with the effective drug concentrations for

B16-F10 cells, we recommend using BA at concentrations of 2.5, 5, and 10 μM; BE at 12.5, 25, and 50 μM; and RAPA at 50 nM for subsequent experiments.

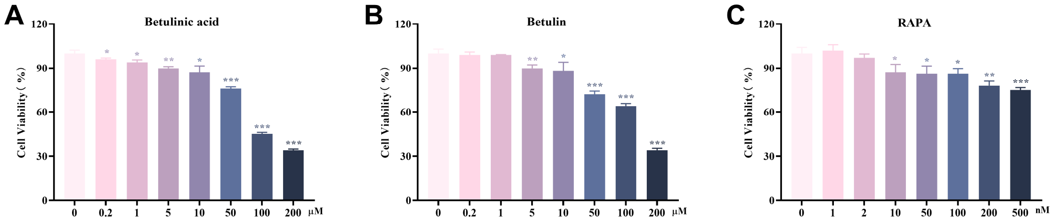

Figure S4. The effect of BA (**A**), BE (**B**) and RAPA (**C**) on the viability of HUVECs cells was determined by the CCK-8 method. * $p < 0.05$; ** $p < 0.01$; *** $p < 0.001$.

2.5 Supplementary Material 5 The effect of BA on organ indices in mice

As shown in Figure S5, compared with the normal group, there was no statistically significant difference in the heart, liver, spleen, lung and kidney indicators of BA-treated mice ($p < 0.05$)

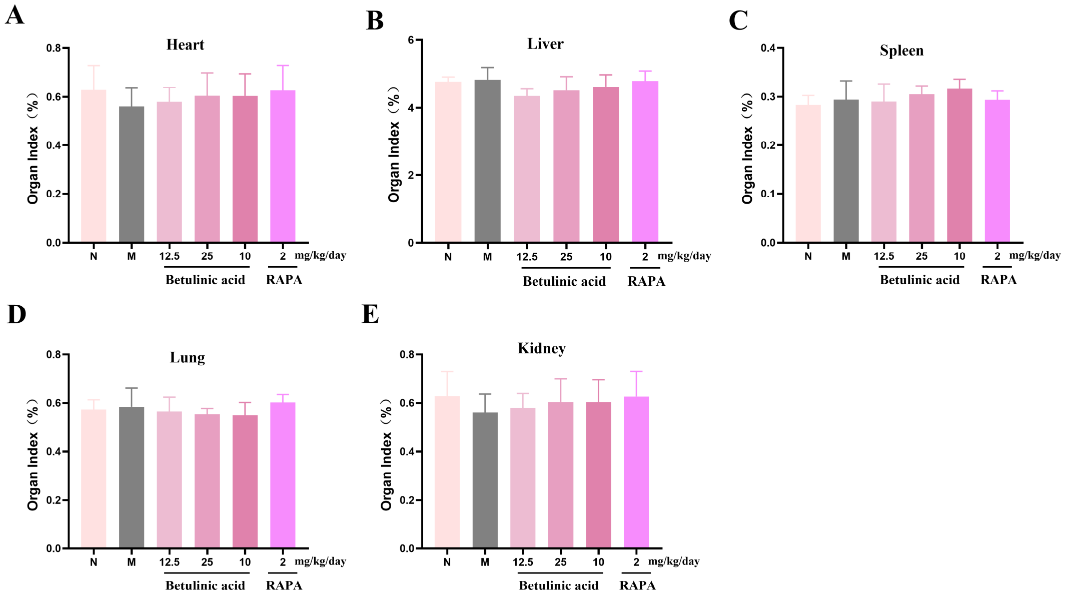

Figure S5. (**A**–**E**) Organ indexes in mice.

2.6 Supplementary Material 6 Quantitative analysis of melanoma tissue pathology

As shown in Figure S6, H&E and TUNEL staining revealed that, in the BA

treatment group, melanin secretion decreased (Figure S6A), and apoptosis increased (Figure S6B).

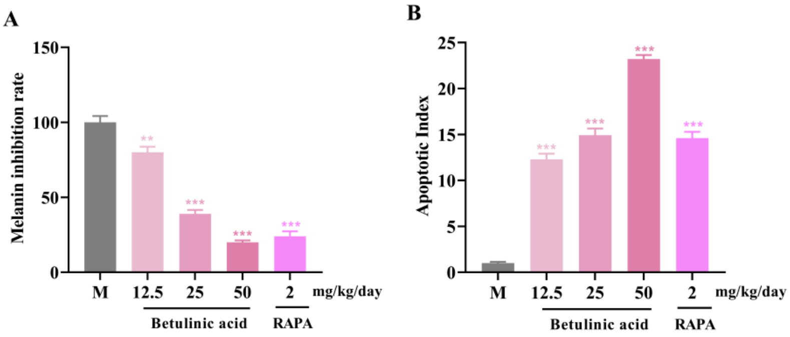

Figure S6. (**A**,**B**) Quantitative analysis of melanoma tissue pathology. ** $p < 0.01$; *** $p < 0.001$.
